# Supplementary material for: Splice-Junction-Based Mapping of Alternative Isoforms in the Human Proteome
Source: Cell Rep. Author manuscript; Available in PMC 2020 Jan 15. (PMC6961840; doi:10.1016/j.celrep.2019.11.026)
Supplement: 2 [file NIHMS1546469-supplement-2.zip › DF1/PXD006675/MitralValve/MitralValve_14_SFXN5_KNHQEGEEAAK.pdf]

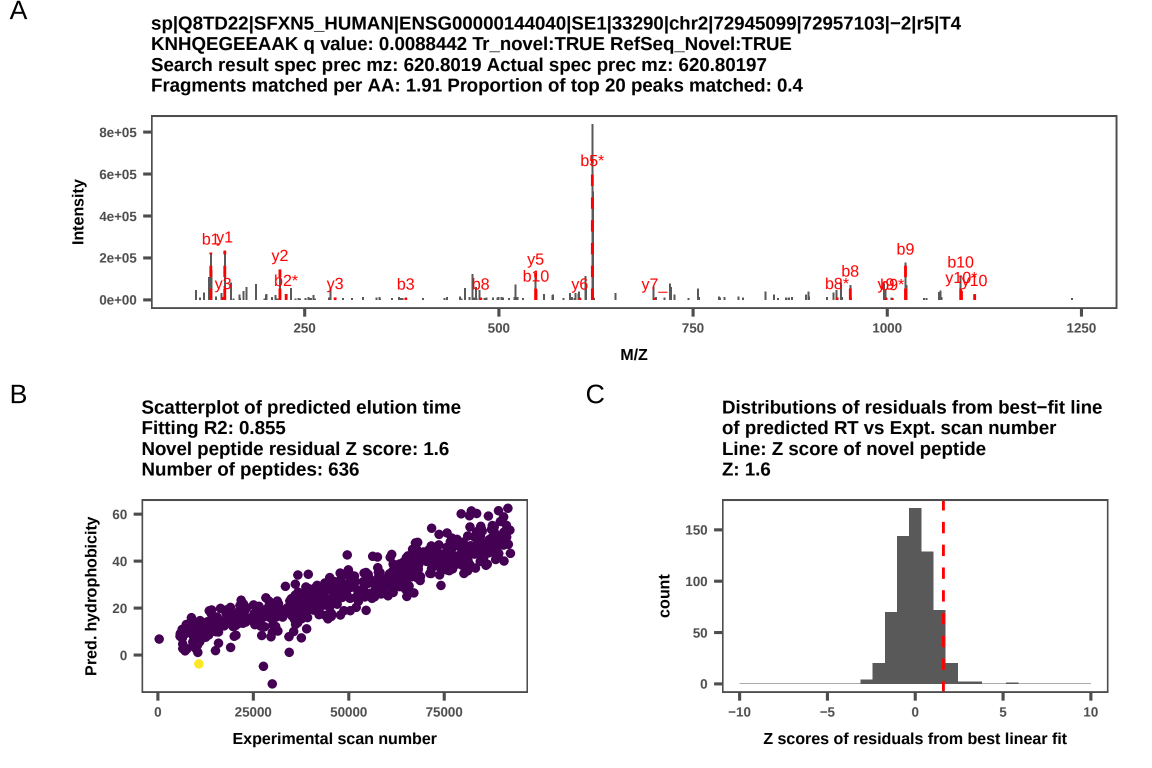
A
sp|Q8TD22|SFXN5_HUMAN|ENSG00000144040|SE1|33290|chr2|72945099|72957103|−2|r5|T4
KNHQEGEEAAK q value: 0.0088442 Tr_novel:TRUE RefSeq_Novel:TRUE
Search result spec prec mz: 620.8019 Actual spec prec mz: 620.80197
Fragments matched per AA: 1.91 Proportion of top 20 peaks matched: 0.4
Intensity
8e+05
6e+05
4e+05
2e+05
0e+00
b1
y1
y3
y2
b2*
y3
b3
b8
y5
b10
y6
b5*
b8*
b8
b9
b10
y10
250
500
750
1000
1250
M/Z
B
Scatterplot of predicted elution time
Fitting R2: 0.855
Novel peptide residual Z score: 1.6
Number of peptides: 636
Pred. hydrophobicity
60
40
20
0
0
25000
50000
75000
Experimental scan number
C
Distributions of residuals from best−fit line
of predicted RT vs Expt. scan number
Line: Z score of novel peptide
Z: 1.6
count
150
100
50
0
−10
−5
0
5
10
Z scores of residuals from best linear fit
